# Laboratory and Field Evaluation of the Phytotoxic Activity of *Sapindus mukorossi* Gaertn Pulp Extract and Identification of a Phytotoxic Substance

**DOI:** 10.3390/molecules26051318

**Published:** 2021-03-02

**Authors:** Ziyang Dai, Jin Wang, Xiaojiang Ma, Jia Sun, Feng Tang

**Affiliations:** SFA Key Laboratory of Bamboo and Rattan Science and Technology, International Centre for Bamboo and Rattan, No. 8 Futong Dongdajie, Wangjing, Chaoyang District, Beijing 100102, China; daiziyang@icbr.ac.cn (Z.D.); mxjicbr@163.com (X.M.); sunjia@icbr.ac.cn (J.S.); fengtang@icbr.ac.cn (F.T.)

**Keywords:** *Sapindus mukorossi*, saponin, phytotoxic activity, root growth inhibition, phytotoxic substance, weed control, field experiment

## Abstract

Interest in finding plant-based herbicides to supplement synthesized herbicides is increasing. Although the extract of *Sapindus mukorossi* Gaertn has been reported to have herbicidal activity, little is known about phytotoxic substances and their efficacy of weed control in the field. To identify phytotoxic substances, the bioassay-guided fractionation by column chromatography and high-speed counter-current chromatography (HSCCC) was carried out. The phytotoxic activity assay, performed by the agar medium method, showed that the 70% ethanol fraction exhibited strong root growth inhibition against *Trifolium pratense* with an 50% inhibitory concentration (IC_50_) value of 35.13 mg/L. An active compound was isolated from the 70% ethanol fraction and identified as hederagenin 3-*o*-*β*-D-xylopyranosyl-(1→3)-α-l-rhamnopyranosyl-(1→2)-α-l-arabinopyranoside (Compound A). Compound A had an IC_50_ value of 16.64 mg/L. Finally, a new formulation was prepared based on the 70% ethanol fraction, which exhibited good efficacy against broadleaf weeds in a carrot field. The fresh weight control efficacy was 78.7% by 45 days after treatment at the dose of 1500 g a. i./ha. Hence, the extract of *S. mukorossi* pulp could be a promising supplement to the synthesized herbicides. Furthermore, compound A from *S. mukorossi* may be responsible for its phytotoxic activity.

## 1. Introduction

Weeds are one of the main factors affecting crops throughout the world as they compete with crops for limited resources [1]. Without weed control, losses of crop yield due to weeds may be up to 70% [2]. Weed control mainly relies on the application of synthesized herbicides. However, overuses of the synthesized herbicides may cause a negative impact on the environment and human health [3]. In addition, hundreds of weeds have evolved resistance to several herbicides [4]. Recently, organic products grown without pesticides have become increasingly popular. Therefore, botanical herbicides will have great development opportunities [5]. 

*Sapindus mukorossi* Gaertn (family: Sapindaceae) is a perennial tree, distributed in sub-tropical and tropical regions [6]. As a medicinal plant, the extract of *S. mukorossi* has been found to possess many biological properties, such as antioxidant [7], antimicrobial [8,9,10,11], antitumor [12], herbicidal [13,14], molluscicidal [15], and insecticidal activities [16]. Various chemical compounds, including triterpenes [12,17], fatty acids [18] and polyphenolic compounds [19] were isolated and identified from *S. mukorossi*. 

Allelochemicals released from plants can affect the weed growth [20]. Different allelochemicals are expected to be developed into natural herbicides [21]. There are several studies about the herbicidal potential of the extract from *S. mukorossi*. The leaf and bark extracts of *S. mukorossi* reveal both inhibitory and stimulatory effects on the germination and root growth of test crops [22]. The leaf extract of *S. mukorossi* has an inhibitory effect on seed germination and root growth in crop pea owing to the presence of allelochemicals [23]. The allelopathic effects may be due to the presence of saponins in *S. mukorossi.* Our preliminary study also verified that the pulp extract of *S. mukorossi* had an inhibition effect on the root growth of *Trifolium pratense* L. and *Cynodon dactylon* L. However, little is known about phytotoxic substances and its efficacy in terms of weed control in the field. 

This study aimed to evaluate the phytotoxic activity of the pulp extract of *S. mukorossi* against *T. pratense* and *C. dactylon*. The bioassay-guided isolation and identification of phytotoxic substances was performed. Furthermore, a new formulation was prepared, and a field test was performed to evaluate the efficacy of the formulation for weed control in carrot. This could lead to the development of a new botanical herbicide.

## 2. Results

### 2.1. Phytotoxicity Screening of the Extract and Fractions from S. mukorossi Pulp

**Figure 1:**
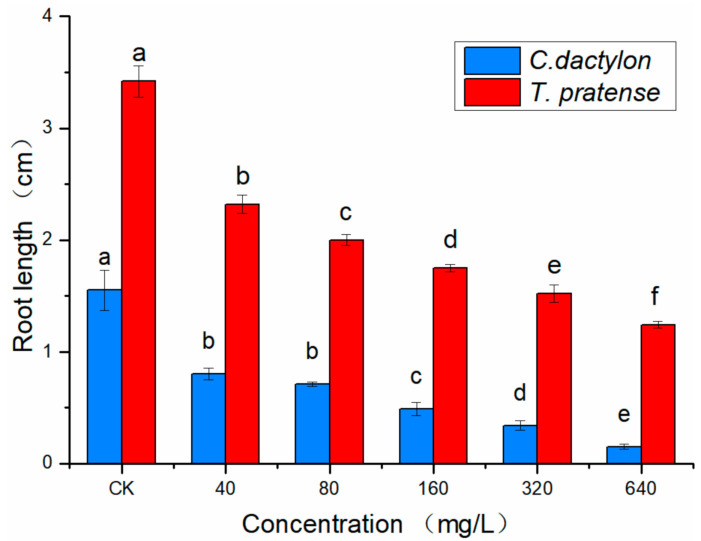
Effect of the ethanol extract of *S. mukorossi* pulp on the root growth of *T. pratense* and *C. dactylon*. The freeze–dried samples in water agar (0.5% agar) were set at 40, 80, 160, 320 and 640 mg/L. CK: untreated plants (control). Root length was recorded after four days of cultivation for *T. pratense* and seven days for *C. dactylon.* Values represent the means ± standard deviation (SD) from three replicates with ten seedlings. Different letters (a, b, c, d, e and f) indicate significant differences among treatments by ANOVA (*p* = 0.05).

To assess the phytotoxic effect of ethanol extract from *S. mukorossi* pulp, root length was recorded. At a concentration of 640 mg/L, the ethanol extract of *S. mukorossi* pulp exhibited the strongest inhibition activity on the root elongation of *T. pratense* and *C. dactylon* (Figure 1). 

**Figure 2:**
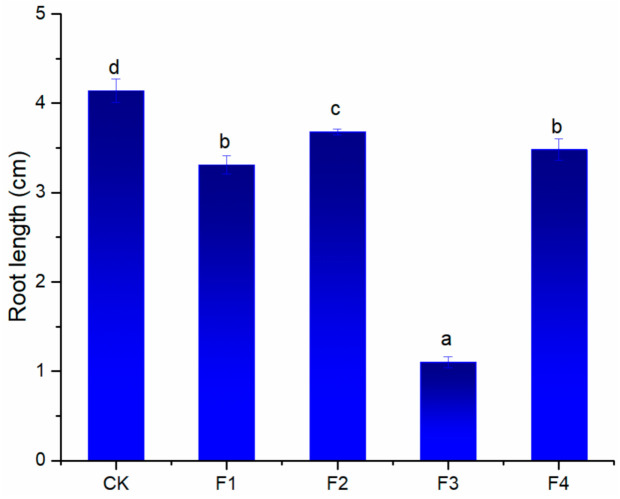
Effect of the fractions obtained from the ethanol extract of *S. mukorossi* pulp against the root growth of *T. pratense* at a concentration of 160 mg/L. CK: untreated plants (control). F1, F2, F3 and F4 represent 30, 50, 70 and 95% ethanol fraction, respectively. Values represent the means ± standard deviation (SD) from three replicates with ten seedlings. Different letters (a, b, c and d) indicate significant differences among treatments by ANOVA (*p* = 0.05).

According to bioassay-guided strategy, the crude extract was further fractionated by macro-porous resin column chromatography with a combination of ethanol and water as eluent. The phytotoxic activity of different samples was evaluated (Figure 2). 

Among the four fractions, the 70% ethanol fraction showed the highest inhibition effect against the root of *T. pratense*. Therefore, the 70% ethanol fraction was selected for further separation using high-speed counter-current chromatography (HSCCC). 

### 2.2. HSCCC Separation and Phytotoxic Activity of Isolated Compounds

**Figure 3:**
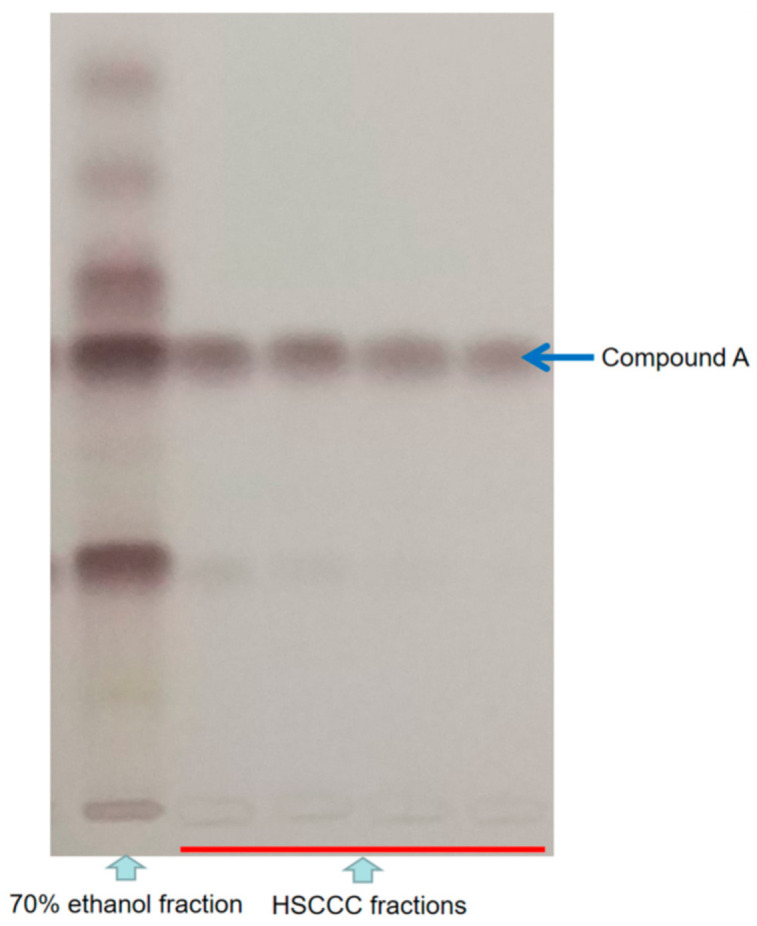
Thin layer chromatography (TLC) photograph of different high-speed counter-current chromatography (HSCCC) fractions and 70% ethanol fraction at white light after derivatization.

To obtain pure compounds, different solvent systems, such as n-hexane-ethyl acetate–methanol–water systems (*v*/*v*/*v*/*v*, 1:3:1:3 or 1:4:1:4 or 1:5:1:5 or 2:5:2:5 or 3:5:3:5), were screened for HSCCC separation. At last, the lower phase of n-hexane-ethyl acetate–methanol–water (1:4:1:4, *v*/*v*/*v*/*v*) was used as the mobile phase and the flow rate was set at 3 mL/min. Under these conditions, a pure fraction (named as compound A) was obtained (Figure 3, see Supplementary Figure S1 for HSCCC spectrum). 

In order to confirm that compound A is one of the effective ingredients, the 70% ethanol fraction and compound A were tested for their half maximal inhibitory concentration (IC_50_) against the root growth of *T. pratense* (Table 1).

As seen in Table 1, compound A exhibited a strong inhibitory activity against the root growth of *T. pratense* with an IC_50_ value of 16.64 mg/L. When compared with 70% ethanol fraction (IC_50_ = 35.13 mg/L), compound A showed 2.11 times more phytotoxic to the target plant *T. pratense*. Therefore, compound A could be one of the effective ingredients.

Compound A was achieved as a white amorphous powder. Its molecular formula was established as C_46_H_74_O_16_ based on a quasi-molecular ion peak at *m*/*z* 883.5053 [M + H]^+^ (calculated value 883.5050). The ^13^C-NMR data of compound A are shown in the Table 2.

**Figure 4:**
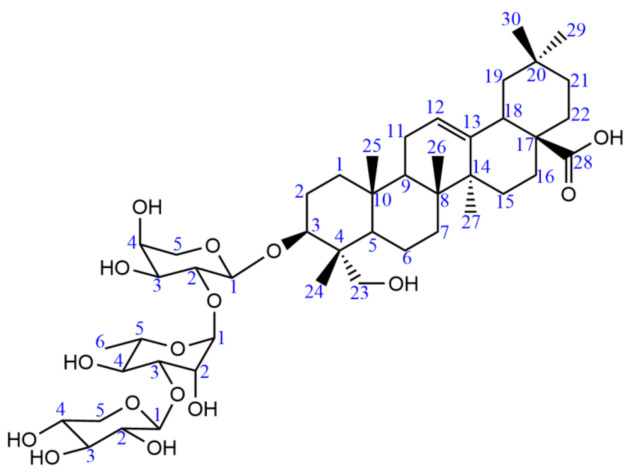
Chemical structure of compound A.

Comparison of the spectral data of compound A with the published literature [24,25], the chemical structure of compound A was determined as hederagenin 3-*o*-*β*-d-xylopyranosyl-(1→3)-α-l-rhamnopyranosyl-(1→2)-α-l-arabinopyranoside (Figure 4).

### 2.3. Field Efficacy of 20% Pulp Extract AS against Weeds in Carrot

The results of the assay in the laboratory need to be verified by field experiment. The 70% ethanol fraction was selected as an active part for formulation preparation and field experiment. Furthermore, a new formulation named as 20% pulp extract AS was prepared and the applied concentrations were 900, 1200 and 1500 g a.i./ha, respectively. The main weed species in the experimental field contained *Eleusine indica* (L.) Gaertn., *Cyperus rotundus* L., *Acalypha australis* L., and *Physalis minima* L. The effect of different treatments on the fresh weight of weeds in carrot is shown in Table 3.

The fresh weight control efficacy of the tested samples varied based on different species of weeds on the 45th day after treatment (DAT). Weed control efficacy of 20% pulp extract AS at a dose of 1500 g a.i./ha showed a significantly pre-emergence inhibition to the broad-leaf weeds, with 88.5% against *A. australis* and 76.7% against *P. minima*, respectively (Table 3). However, the control effect on the narrow-leaved weed *E. indica* was poor (varied between 25.6 and 69.6%) at both low and high doses. The total efficacy of 20% pulp extract AS at a dose of 1500 g a.i./ha was 78.7%. As for some weeds (such as *C. rotundus* and *A. australis*), 20% pulp extract AS was more effective in the fresh weight control than positive control herbicide. Meanwhile, the control efficacy according to the weed population was also calculated. The data regarding the weed control at 30 and 45 DAT are listed in Table 4.

As shown in Table 4, the results indicated that all the treatments significantly reduced the weed population in carrot field compared to the blank control. It can also be seen that the control efficacy of 20% pulp extract AS against weeds was dose-dependent. The highest weed control efficacy (72.2%) was recorded at a dose of 1500 g a.i./ha. Therefore, the recommended dose of 20% pulp extract AS was equal or more than 1500 g a.i./ha for weed control in carrot field.

The purpose of using herbicides is to reduce the biomass and density of weeds in the field. The fresh weight control efficacy is to characterize the effect of herbicides on weed biomass (see Table 3). The weed control efficacy is to characterize the effect of herbicides on weed density (see Table 4). Based on the results of Table 3 and Table 4, 20% pulp extract AS at a dose of 1500 g a.i./ha significantly decreased weed density and biomass in the carrot field. 

## 3. Discussion

As an ecological phenomenon, some plants affect the growth of neighboring plants by releasing allelopathic substances into the environment [26,27]. Recent studies have revealed that the extracts of *S. mukorusii* can inhibit the growth and germination of tested plants, and several active compounds (such as oleanolic acid, lupeol, d-pinitol, hexadecanoic acid and octadecanoic acid) have been identified in the leaf extract [13,27]. The volatiles from the pericarp of *S. mukorossi* can inhibit radicle elongation (3% of control) of lettuce [28].

In this study, the pulp extract and its fractions significantly inhibited the root elongation of *T. pratense* and *C. dactylon*. A new AS formulation was developed without any organic solvent and considered as a more safe and environmentally friendly formulation than the EC formulation. The developed pulp extract AS was successfully applied in carrot field has not been reported in the literature. Weed control is a challenge in vegetable production because few herbicides have broad-spectrum weed control and crop tolerance [29]. The pre-emergence application of 20% pulp extract AS at 45 days after treatment was very effective against broad-leaf weeds. However, compared to synthetic herbicide (33% pendimethalin EC), 20% pulp extract AS performed worse in controlling narrow-leaf weeds such as *E. indica.* It would be better that herbicide combined pendimethalin and *S. mukorossi* extract for broad spectrum weed control in carrot.

Different weeds were observed in the field, and there were differences in their susceptibility to the herbicides. Compared to synthetic herbicide (33% pendimethalin EC), 20% pulp extract AS performed better in controlling *C. rotundus* and *A. australis*. Maybe, the two weeds mentioned above were more susceptible to 20% pulp extract AS than 33% pendimethalin EC. 

The plant of *S. mukorossi* has been used as traditional medicine in Asian countries for a long time [30]. In addition, the extract from *S. mukorossi* has been proved to be safe for cosmetic use [31,32]. Therefore, the pulp extract of *S. mukorossi* could be safe compared to the synthesized herbicide. Nevertheless, the safety and the herbicidal action mechanism of the developed formulation needs to be further studied. 

In conclusion, the 70% ethanol fraction obtained from the crude extract of *S. mukorossi* pulp exhibited a strong inhibitory effect against the growth of *T. pratense*. An active compound was isolated and identified as hederagenin 3-*o*-*β*-d-xylopyranosyl-(1→3)-α-l-rhamnopyranosyl-(1→2)-α-l-arabinopyranoside (Compound A). Furthermore, a novel botanical herbicide formulation was prepared and successfully applied in carrot field for weed control. 

## 4. Materials and Methods

### 4.1. Material and Reagents

The fruits of *S. mukorossi* were collected at the foot of Dashu Mountain (31.849° N, 117.177° E, and elevation 53 m), Hefei, Anhui Province, China in November 2017 and authenticated by Prof. Yimin Hu from the Anhui Academy of Forestry, China. A voucher specimen was deposited at the key Laboratory of Bamboo and Rattan Science and Technology. The fruits were dried in the shade at room temperature and the pulp of the fruits was manually separated from the seeds. The dried pulp was ground into powder and stored at −20 °C until further use.

Methanol (HPLC grade) was purchased from Fisher Scientific (Fair Lawn, NJ, USA). Agar was obtained from BioFroxx GmbH (Einhausen, Hessen, Germany). AB-8 macroporous resin (pore size 13–14 nm) was purchased from Tianjin Nankai Hecheng Science & Technology Co., Ltd. (China). All the other reagents were of analytical grade.

### 4.2. Apparatus

The preparative HSCCC instrument was a TBE-300B (Tauto Biotech, Shanghai, China), which was equipped with a model TBP-5002 constant-flow pump. ESI–MS spectra were obtained with a liquid chromatography/quadrupole time-of-flight mass spectrometry (model 6540, Agilent Technologies, Santa Clara, CA, USA). NMR analysis was performed using a Bruker Avance 600 NMR spectrometer (Bruker, Karlsruhe, Germany) with pyridine-*d*_5_ as solvent. 

### 4.3. Preparation of Pulp Extract of S. mukorossi

The dried powdered pulp (5 kg) was extracted three times with 95% ethanol (25 L) at room temperature. The combined ethanol extract was filtered and evaporated in a rotary evaporator at 45 °C. The extract was freeze–dried, yielding a yellow-brown solid (1850 g). A portion (1080 g) of the freeze–dried extract was loaded onto a macroporous resin AB-8 column chromatography (105 cm × 12 cm i.d.), eluted with water–ethanol (100:0, 70:30, 50:50, 30:70, 5:100, *v*/*v*). The collected fractions were evaporated and freeze–dried, yielding a 30% ethanol fraction (100 g), 50% ethanol fraction (259 g), 70% ethanol fraction (187 g) and 95% ethanol fraction (75 g).

### 4.4. HSCCC Separation and TLC Analysis

The two-phase solvent system composed of n-hexane-ethyl acetate–methanol–water (*v*/*v*/*v*/*v*, 1:4:1:4) was selected for HSCCC. The upper stationary phase was pumped into the coiled HSCCC column at 20 mL/min. Then, the apparatus was rotated at 900 rpm, and the lower phase was pumped into the column at 3 mL/min. After a hydrodynamic equilibrium was reached, about 10 mL sample solution containing 50 mg of 70% ethanol fraction was injected. The HSCCC fractions were collected using a fraction collector set at 5 mL for each tube. The running time was ~150 min for each sample. Different fractions were detected by thin layer chromatography (TLC). Thin-layer chromatography was carried out on the plates precoated with silica gel 60 F254 (Merck, Darmstadt, Hessen, Germany). Samples (2 μL) were applied to the plate using a Camag (Switzerland) Linomat V sample applicator. The plate was developed using n-butanol: water: acetic acid (84:14:7, *v*/*v*/*v*) as the developing solvent [33]. The developed plates were derivatized by immersing with ethanol: sulphuric acid (90:10, *v*/*v*) followed by heating at 110 °C for 10 min [34]. A pure fraction (named as compound A) was obtained.

### 4.5. Phytotoxic Activity in Laboratory

Phytotoxic activity was evaluated using the seed germination method in Petri plates as previously described [13]. Two plants (*T. pratense* and *C. dactylon*) were used as target plants. The seeds were sterilized in 0.2% sodium hypochlorite. The freeze–dried samples in water agar (0.5 % agar) were set at 40, 80, 160, 320 and 640 mg/L. Pure water was used as a blank control. The seeds were sown in 9 cm diameter Petri dishes (10 seeds per dish). Root length was recorded after four days of cultivation for *T. pratense* and seven days for *C. dactylon*. Because there was not enough pure compound A, miniaturized bioassay in 24 wells (5 seeds per well) was carried out [29]. The assay concentrations were 5, 10, 20, 40, 80 and 160 mg/L for compound A and 10, 20, 40, 80, 160 and 320 mg/L for 70% ethanol fraction, respectively. Three replicates were performed for each treatment. 

### 4.6. Formulation Preparation and Its Field Test

The 70% ethanol fraction, as an active ingredient, was selected for formulation preparation. An optimized aqueous solution (AS) formulation was prepared, which was composed of 70% ethanol fraction (20%, *g*/*v*), emulsifier (4%, g/*v*), water (73%, *v*/*v*) and antifreeze (3%, *v*/*v*). The evaluation of physical properties of the developed formulation (20% pulp extract AS) was carried out. According to the HG/T2467.6-2003 standard, the dilution stability and the stability at low temperature (0 °C, 7 days) and thermal storage (54 ± 2 °C, 14 days) of the developed formulation were determined. 

The filed experiment of the pulp extract formulation against weeds was carried out in a carrot (*Daucus carota* L.) field in 2019 in the Xiaoxian County of Anhui province, China. The soil was sandy loam and slightly alkaline (pH 7.6). The field experiment was carried out according to the national test guidance in China (GB/T 17980.47-2000 standard). The experiment field was divided into 15 plots and each plot was 10.5 m^2^ (3.5 m × 3 m) in size. The experiment was designed with five treatments and three replications with random assignment. The developed formulation was set at 900 g a.i./ha, 1200 g a.i./ha and 1500 g a.i./ha, respectively. One chemical herbicide, 33% pendimethalin EC (at the recommended dose of 742.5 g a.i./ha), was selected as a positive control. Pure water was chosen as a blank control. The plots were sprayed at pre-emergence stage (1 day after sowing) with a spray volume of 694 L/ha. The data on weed count and weed fresh weight were recorded from three 0.25 m2 quadrats in each plot at 30 and 45 days after treatment, respectively. Control efficacy (%) of the developed formulation AS and the pendimethalin EC against weeds in carrot field were evaluated according to the equation [13]:

Control efficacy (%) = (G − G_1_)/G × 100 (1)

where G is the weed count or fresh weight of the weeds in the control plot, and G_1_ is the weed count or fresh weight of the weeds in treatment plot.

### 4.7. Statistical Analysis

IC_50_ was evaluated using probit method. SPSS software (version 26, IBM Corp., Armonk, NY, USA) was used for statistical analysis. One-way ANOVA followed by Duncan’s test was used to calculate significant differences with a significant level at *p* = 0.05.

## Figures and Tables

**Table 1:** Root growth inhibition activity of different samples against *T. pratense*. Data are presented as 50% inhibitory concentration (IC_50_) together with its respective 95% confidence intervals (95% CI). Data presented are the mean of three replicates. IC_50_ value was obtained using log-probit analysis.

Sample	IC_50_ (95% CI) (mg/L)	Slope ± SE	χ^2^
Compound A	16.64 (12.34–21.47)	0.97 ± 0.11	2.39
70% ethanol fraction	35.13 (25.29–46.40)	0.87 ± 0.11	1.37

**Table 2:** ^13^C-NMR data of compound A in pyridine-d5 compared with literature data.

C	DEPT	Compound A	Reference	C	DEPT	Compound A	Reference
1	CH_2_	39.4	39.3	26	CH_3_	17.8	17.9
2	CH_2_	26.7	26.5	27	CH_3_	26.5	26.4
3	CH	81.4	81.3	28	-	180.9	182.0
4	-	43.9	43.8	29	CH_3_	33.7	33.1
5	CH	47.9	47.9	30	CH_3_	24.1	23.9
6	CH_2_	18.5	18.4				
7	CH_2_	33.2	33.2	Arabinose			
8	-	40.0	40.0	1	CH	105.0	104.3
9	CH	48.5	48.5	2	CH	75.9	75.4
10	-	37.2	37.2	3	CH	75.4	74.8
11	CH_2_	24.2	24.0	4	CH	69.8	69.4
12	CH	122.8	122.6	5	CH_2_	66.6	65.9
13	-	145.2	145.5	Rhamnose			
14	-	42.4	42.4	1	CH	101.6	101.1
15	CH_2_	28.6	28.7	2	CH	72.3	71.7
16	CH_2_	24.0	24.0	3	CH	82.8	82.6
17	-	46.7	46.9	4	CH	72.7	72.7
18	CH	42.5	42.4	5	CH	69.9	69.4
19	CH_2_	46.7	47.0	6	CH_3_	18.7	18.3
20	-	31.3	31.2	Xylose			
21	CH_2_	34.5	34.6	1	CH	107.8	107.1
22	CH_2_	33.6	33.6	2	CH	75.5	75.2
23	CH_2_	64.3	64.4	3	CH	78.7	78.1
24	CH_3_	14.5	14.4	4	CH	71.4	70.8
25	CH_3_	16.4	16.4	5	CH_2_	67.7	67.1

**Table 3:** Control efficacy of 20% pulp extract AS and pendimethalin EC against the weed fresh weight in carrot at 45 days after treatment. Data presented are the mean of three replicates ± standard deviation (SD). “Total” represents the fresh weight control efficacy for all weeds in carrot field.

Treatment	Dose (g a.i./ha)	Fresh Weight Control Efficacy (%)				
		*Eleusine indica* (L.) Gaertn.	*Cyperus rotundus* L.	*Acalypha australis* L.	*Physalis minima* L.	Total
20% pulp extract AS	1500.0	69.6 ± 2.5	83.9 ± 3.9	88.5 ± 5.2	76.7 ± 3.7	78.7 ± 1.0 ^b^
	1200.0	50.0 ± 4.7	63.4 ± 3.7	67.7 ± 4.3	60.5 ± 5.1	61.5 ± 2.8 ^c^
	900.0	25.6 ± 3.4	37.7 ± 4.5	41.1 ± 4.7	49.0 ± 3.6	28.2 ± 1.7 ^d^
33% pendimethalin EC	742.5	100.0 ± 0.0	42.5 ± 4.5	65.4 ± 4.0	99.7 ± 0.5	93.4 ± 0.8 ^a^
Blank control (water)	0	0	0	0	0	0 ^e^

(a, b, c, d and e) in the same column are significantly different in an ANOVA test (*p* = 0.05).

**Table 4:** Field efficacy of 20% pulp extract AS and pendimethalin EC against weeds in carrot.

Treatment	Dose (g a.i./ha)	Weed Control Efficacy (%)	
		30 DAT	45 DAT
20% pulp extract AS	1500.0	70.1 ± 2.8 ^b^	72.2 ± 2.4 ^b^
	1200.0	53.1 ± 2.7 ^c^	59.7 ± 2.9 ^c^
	900.0	39.0 ± 3.4 ^d^	45.0 ± 2.1 ^d^
33% pendimethalin EC	742.5	84.8 ± 0.98 ^a^	88.3 ± 1.4 ^a^
Blank control (water)	0	0 ^e^	0 ^e^

Mean labeled with different letters (a, b, c, d and e) in the same column are significantly different in an ANOVA test (*p* = 0.05). DAT: days after treatment. Data presented are the mean of three replicates ± standard deviation (SD).

## Data Availability

Not applicable.

## Supplementary Materials

The following are available online. Figure S1: High-speed counter-current chromatography (HSCCC) spectrum of the 70% ethanol fraction. Peak of compound A is marked with red arrow. Figure S2: ESI-MS spectrum of compound A obtained by LC-QTOF/MS in the positive ion mode.

## Sample Availability

Samples are available from the authors.
