# Supplementary material for: Laboratory and Field Evaluation of the Phytotoxic Activity of Sapindus mukorossi Gaertn Pulp Extract and Identification of a Phytotoxic Substance
Source: Molecules. 2021 Mar 2;26(5):1318. doi: 10.3390/molecules26051318 (PMC7957876; doi:10.3390/molecules26051318)

Figure S1: High-speed counter-current chromatography (HSCCC) spectrum of the 70% ethanol fraction. Peak of compound A is marked with red arrow.

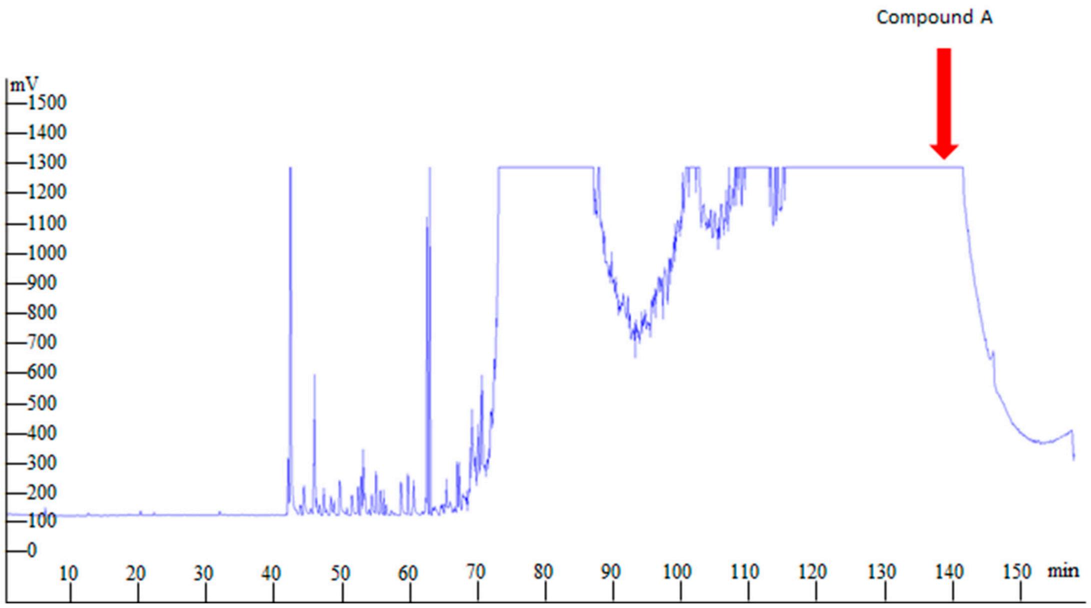

Figure S2: ESI-MS spectrum of compound A obtained by LC-QTOF/MS in the positive ion mode

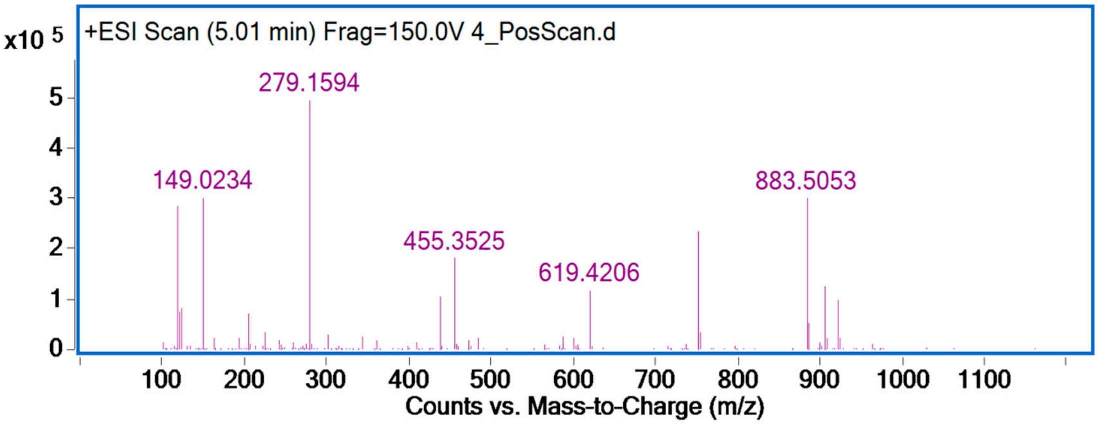

Supplement: Supplementary file 1 [file molecules-26-01318-s001.pdf]
